# African Swine Fever Virus Isolate, Georgia, 2007

**DOI:** 10.3201/eid1412.080591

**Published:** 2008-12

**Authors:** Rebecca J. Rowlands, Vincent Michaud, Livio Heath, Geoff Hutchings, Chris Oura, Wilna Vosloo, Rahana Dwarka, Tinatin Onashvili, Emmanuel Albina, Linda K. Dixon

**Affiliations:** Institute for Animal Health, Pirbright, UK (R.J. Rowlands, G. Hutchings, C. Oura, L.K. Dixon); Centre de Coopération Internationale en Recherche Agronomique pour le Développement, Montpellier, France (V. Michaud, E. Albina); Agricultural Research Council–Onderstepoort Veterinary Institute, Onderstepoort, South Africa (L. Heath, W. Vosloo, R. Dwarka); University of Pretoria, Pretoria, South Africa (W. Vosloo); Laboratory of Ministry of Agriculture of Georgia, Tbilisi, Georgia (T. Onashvili)

**Keywords:** African swine fever virus, genome analysis, transmission, Georgia, research

## Abstract

African swine fever (ASF) is widespread in Africa but is rarely introduced to other continents. In June 2007, ASF was confirmed in the Caucasus region of Georgia, and it has since spread to neighboring countries. DNA fragments amplified from the genome of the isolates from domestic pigs in Georgia in 2007 were sequenced and compared with other ASF virus (ASFV) isolates to establish the genotype of the virus. Sequences were obtained from 4 genome regions, including part of the gene *B646L* that encodes the p72 capsid protein, the complete *E183L* and *CP204L* genes, which encode the p54 and p30 proteins and the variable region of the *B602L* gene. Analysis of these sequences indicated that the Georgia 2007 isolate is closely related to isolates belonging to genotype II, which is circulating in Mozambique, Madagascar, and Zambia. One possibility for the spread of disease to Georgia is that pigs were fed ASFV-contaminated pork brought in on ships and, subsequently, the disease was disseminated throughout the region.

## Abstract 2

The virus isolate introduced to the Caucasus in 2007 is closely related to a group of viruses, genotype II, circulating in Mozambique, Madagascar, and Zambia.

African swine fever (ASF), classified as a notifiable disease by the World Organisation for Animal Health (OIE), causes an acute hemorrhagic fever in domestic pigs. It often results in major economic losses because of the high rates of illness and death associated with the disease. ASF has the potential to spread rapidly and since a vaccine is currently not available, control options are limited to rapid diagnosis of the disease and culling of infected animals and animals in contact with them.

ASF virus (ASFV) infects wildlife hosts and ticks of *Ornithodoros* spp*.*, and these can provide a reservoir of virus that is not possible to eliminate. In Africa, where ASF is widespread, the virus causes long-term, persistent infections—but no clinical manifestation of disease—in warthogs (*Phacochoerus africanus*) and bushpigs (*Potamochoerus porcus*) (*1*). In contrast, ASFV causes clinical disease and high numbers of deaths in wild boars (*Sus scrofa*). The disease has been reported in pigs from most sub-Saharan countries and continues to spread to previously uninfected countries within the region. In 1998, ASF was reported in Madagascar for the first time; it is now considered to be endemic. At the end of 2007, ASF was introduced on a second Indian Ocean island, Mauritius (*2*).

Once introduced into countries, ASF is difficult to eradicate for several reasons, including the presence of wildlife reservoirs, lack of a vaccine, insufficient laboratory support for rapid and accurate diagnosis, and inadequate funding for veterinary services to enforce the appropriate control measures. This situation was amply demonstrated in Portugal and Spain, where the disease remained endemic until the 1990s, after its introduction into Portugal in 1957 and again in 1960. Other European countries, the Caribbean, and Brazil have had outbreaks of ASF, but extensive control programs have led to successful eradication, with the exception of Sardinia, where ASF has remained endemic since 1982 (*1*).

The genetic characterization of the viral strain associated with disease outbreaks is important for tracing possible sources of infection and ensuring that appropriate diagnostic reagents are used. ASFV isolates have previously been characterized by restriction enzyme site mapping or sequencing of different genome regions. Partial sequencing of the *B646L* gene encoding the major capsid protein p72 has so far led to the identification of 22 ASFV genotypes. Twenty-one of these genotypes were identified in isolates from domestic pigs or from wildlife hosts in eastern and southern Africa. The level of diversity between isolates from these regions is attributed to the long-term evolution of virus within wildlife hosts. In contrast to the other genotypes, genotype I predominantly comprises isolates from domestic pigs in West and Central Africa, Europe, the Caribbean, and Brazil obtained during a 40-year period since 1957. Isolates belonging to genotype I share considerably higher sequence identity across the p72 gene compared to isolates from the sylvatic cycle, which suggests that this genotype probably evolved from a single source introduction (*3*–*5*). PCR amplification and sequencing of more variable genome regions have been used to distinguish between closely related isolates and identify virus subgroups within several of the 22 genotypes (*4*). The additional genome regions that have been described thus far include the *E183L* and *CP204L* gene regions, encoding the p54 and p30 proteins, respectively, as well as the central variable region within the open reading frame (ORF) *B602L*.

ASFV can infect pigs by a variety of mechanisms, including direct contact between pigs, bites from infected ticks, indirect transmission by means of fomites, and ingestion of infected meat. The main route by which ASF infections spread over long distances is thought to be infected meat products. The transcontinental spread of ASF has been a relatively rare event, and it was unexpected when, in June 2007, cases of ASF affecting domestic pigs in the Caucasus region of the former Soviet republic of Georgia were confirmed by the OIE ASF reference laboratory (*6*). It has been suggested that the outbreak started in April 2007 near the Black Sea Port of Poti. Catering waste, including infected pig meat from ships in the port, is considered to be the most likely source of the infection. As of July 9, 2007, the outbreak had spread to 56 of 61 districts in Georgia. Reports to OIE indicated that >80,000 pigs had died or been destroyed in Georgia. Outbreaks of ASF were also reported in neighboring regions, including the autonomous republic of Abkhazia (*7*). On August 29, 2007, ASF was confirmed in Armenia and on November 4, 2007 (*8*), in Nagorno-Karabakh (*9*), a de facto independent republic that is officially part of Azerbaijan and near its border with Armenia. On November 5, 2007, infection of a wild boar was confirmed in the Russian Republic of Chechnya near the border with Georgia. To control the spread of disease, wild boars were killed in 17 different regions in Chechnya, and the slaughter of the entire pig population was ordered (*10*). Further outbreaks of ASF were reported in Nagorno-Karabakh in April 2008, where it is believed that ≈8,500 pigs have died as a result of disease since the beginning of the outbreaks.

Here we describe the genetic characterization of the ASFV isolates implicated in the 2007 outbreak of the disease in Georgia. Results of the analysis showed that the Georgia isolates group within genotype II, which suggests that the virus is closely related to ASFV isolates typically found in Mozambique, Madagascar, and Zambia (*4*,*11*,*12*).

## Materials and Methods

### Virus Isolates

In June 2007, samples were collected from 2 pigs that were showing clinical signs of ASF. The first pig originated from the Imereti Province in western Georgia; the second was sampled in the Kakheti Province in eastern Georgia. Five tissues samples were collected from each pig, including serum and samples from the kidney, spleen, lung, and lymph nodes. The samples were subsequently submitted to the OIE reference laboratory, Institute for Animal Health, Pirbright, United Kingdom. The presence of ASFV in these samples was confirmed by pathogen isolation on primary leukocyte cultures, real-time PCR, and ELISA (*13*,*14*).

### Viral DNA Extraction, PCR Amplification, and Sequencing

Viral DNA was extracted directly from cell culture isolates or from suspensions of clinical samples by using the High Pure Viral Nucleic Acid Kit (Roche, Indianapolis, IN, USA) following the manufacturer’s guidelines. The extracted DNA was used as template for the amplification of the respective gene regions. Details of isolates studied are shown in the Appendix Table.

PCRs were performed with the Accuprime *Pfx* DNA polymerase (Invitrogen, Carlsbad, CA, USA). Reactions contained 22.5 μL Accuprime *Pfx* Supermix, 100 ng DNA, and a final concentration of 200 nmol/L of each primer in a total reaction volume of 25 μL. Thermocycling condition included a 2-min denaturation step of 95°C, followed by 35 cycles of 30 s at 95°C, 30 s at 60°C, and 30 s at 68°C with a 10-min elongation step at 68°C. Part of the gene encoding the p72 gene was amplified by using the primers P72-D and P72-U (*3*), which amplify a 478-bp fragment from the 3′ end of the *B646L* gene. The primer pair ORF9L-F (5′-AATGCGCTCAGGATCTGTTAAATCGG-3′) and ORF9L-R (5′-TCTTCATGCTCAAAGTGCGTATACCT-3′) was used to amplify a region from the central variable genome within the ORF B602L (*16*); E183L-F (5′-TCACCGAAGTGCATGTAATAAACG-3′) and E183L-R (5′-TCTGTAATTTCATTGCGGCCACAACATT-3′) were used to amplify a 681-bp fragment of the *E183L* gene. Primer pairs p30-F (5′-ATGAAAATGGAGGTCATCTTCAAAAC-3′) and p30-R (5-AAGTTTAATGACCATGAGTCTTACC-3′) were used to amplify 521 bp of the *CP204L* gene.

Primers used for the amplification of p72, p54, p30, and B602L gene regions, as described above, were used in the respective sequencing reactions. Sequencing of PCR products was performed by using the Dye Terminator Cycle Sequencing Quick Start Kit (Beckman Coulter, Fullerton, CA, USA). Thermocycling consisted of 30 cycles of 96°C for 20 s, 50°C for 20 s, and 60°C for 3 min. Completed reactions were processed following the manufacturer’s instructions. Data was processed by using the default sequence analysis parameters and analyzed with Beckman Coulter CEQ 8000 software.

#### Sequence Analysis

Analysis of sequence data was performed with Beckman Coulter CEQ8000 software, Chromas (www.technelysium.com.au), BioEdit (www.mbio.ncsu.edu/BioEdit/BioEdit.html), and ClustalX version 1.83 (www.clustal.org). A summary of the sequences is shown in the Appendix Table.

Phylogenetic analysis was conducted by means of the “criterion of neighborhood based on the principle of parsimony” (www.megasoftware.net/index.html; *17**,**18*), selecting the correction of Kimura (*19*). Bootstrap confidence values were calculated on 1,000 replicates according to the maximum likelihood approach of Felsenstein (*20*).

## Results

### Partial Sequence of *B646L* Gene Encoding the p72 Capsid Protein

Sequence analysis of the *B646L* gene has been used extensively for phylogenetic analysis of ASFV isolates (*3*,*5*,*15*) by focusing on a 478-bp fragment corresponding to the C-terminal end of the *B646L* gene that broadly defines the virus genotypes. Twenty-two genotypes (*4*) have thus far been identified by analyzing this region of the viral genome.

The *B646L* partial sequences from each of the 5 tissue samples from the east and west Georgian samples showed that they were identical at the nucleotide level (results not shown). Comparison of these sequences to other isolates of known genotypes identified the Georgia 2007 sequence as falling within *B646L* genotype II (Figure), together with 1 isolate from Zambia (Lus 1/93), isolated from a domestic pig after an outbreak of ASF in 1991 (*10*); 9 from Mozambique (Moz 60–98, Moz 61–98, Moz 63–98, Moz 70–98, Moz 77–98, Moz 1/02, Moz 2/02, Moz 1/03, and Moz 1/05), obtained from outbreaks in 1998–2005 (*5*,*11*,*12*); and a pig isolate from Madagascar (Mad 1/98), obtained after the first introduction in 1998 (*3*,*21*).

**Figure:**
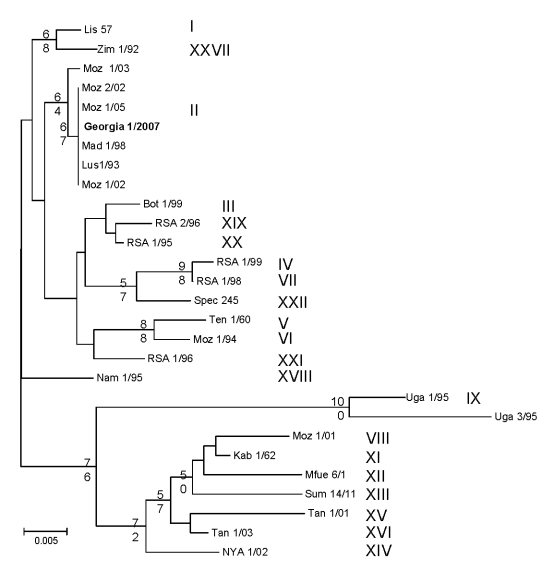
Phylogram depicting the *B646L* gene relationships of selected isolates representative of the 22 African swine fever virus genotypes. Because all the Georgian isolates had identical nucleotide sequences, only 1 isolate is presented in the tree (in **boldface**). The consensus tree was generated from 1,000 replicates; only bootstraps >50% are shown. Genotypes are indicated in roman numerals. Moz, Mozambique. Scale bar indicates number of nucleotide substitutions per site.

### Sequence Analysis of *B602L* Region

The central variable region of the ORF *B602L* is characterized by tetrameric repeats, the number and composition which can be used to distinguish between closely related isolates (*16*). Sequence analysis of this region from the *B602L* gene (also designated central variable region ORF9L, 9RL) of >100 ASFV isolates has shown that the number of tandem repeat tetramers in individual genomes may vary from 7 to 34. Twenty-two sequence variants of the 4-aa repeats have also been identified (*15*).

Amplification of the *B602L* variable fragment from each of the east and western Georgian isolates yielded PCR products of ≈200 bp, which corresponded in size and sequence to the other genotype II isolates with 10-aa tetramers. The sequences of this region differed from that of all other genotypes (Appendix Figure 1). Despite also containing 10 copies of amino acid tetramers, the *B602L* sequence of 2 South African isolates from genotype XXI differed from Georgia 2007 and the other genotype II isolates.

### Sequence Analysis of *E183L* Gene Encoding Protein p54

Amplification of the fragment containing the complete *E183L* gene from all the Georgian isolates produced PCR products of ≈550 bp, which were identical in sequence (results not shown). Amplification of this fragment from other isolates from Africa, Europe, and Madagascar produced fragments that ranged from 528 bp to 600 bp. The discrepancy in the size of the respective fragments is due to sequences encoding 2 arrays of amino acid repeats, which vary in number and sequence. The nucleotide sequence of the *E183L* gene from the Georgia 2007 isolate was identical to sequences from 5 Madagascar isolates obtained from outbreaks that in 1998–2003 (Mad 1/98, Ampani/99, Tolagna/99, Chrome/01, and Antani/03) and 2 Mozambican isolates (Moz 1/02, Moz 2/02) (Appendix Figure 2). However, the p54 nt and protein sequences of isolates Moz 1/03, Moz 1/05, and Lus 1/93 differed from that of the Georgia 2007 isolates; the latter contained a single deletion between positions 341 and 355, resulting in a 5-aa deletion within the central portion of the protein. The nucleotide sequence of the 2 other isolates from Mozambique obtained in 2003 and 2005 (Moz 1/03 and Moz 1/05) were identical to each other but differed from that of the Georgia 2007 isolates at several positions throughout the gene region (Appendix Figure 2).

### Sequence Analysis of *CP204L* Gene Encoding p30 Protein

Amplification of a fragment containing the *CP204L* gene from each of 2 Georgian isolates produced a PCR product of ≈550 bp. As was the case in all the other gene regions, the sequence of the 2 Georgian isolates was identical across the length of the gene (results not shown). The nucleotide sequences of the Georgia isolates were unique within genotype II but shared a high degree of similarity with the isolates from Madagascar, Zambia, and Moz 2/02 (nucleotide identity >99%). In contrast, isolates implicated in the most recent outbreaks of the disease in Mozambique, isolates Moz 1/03 and Moz 1/05, differed from the Georgian isolates by >2.5% at the nucleotide level (Appendix Figure 3).

## Discussion

We analyzed the sequence of 4 genomic fragments of the ASFV genome to characterize the viruses responsible for the outbreak of ASF in Georgia in 2007. The 4 regions of the genome—*B646L, E183L, CP204L,* and the variable region within the ORF *B602L*—were amplified by PCR. The nucleotide and amino acid sequences of these ORFs from samples collected at 2 different geographic locations in Georgia were then compared with ASFV isolates from other regions of the world. Because all DNA and amino acid sequences for each genome region from all tissue samples obtained from Georgia that we tested were identical, we concluded that the ASF outbreaks in Georgia and the surrounding regions were probably due to a single introduction of the virus. Sequence analysis of the p72 gene region placed the Georgian isolate within genotype II together with isolates from Madagascar, Mozambique, and Zambia (*3*–*5*). Genotype II occurred in Mozambique in outbreaks in 1998–2005 (*12*) and affected the northeastern provinces of Cabo Delgado and Nampula (which were most recently affected in 2004), the northwestern province of Tete, and the southern province of Maputo (most recently in 2005) (*12*). Three other genotypes of ASFV have also been identified as having occurred in Mozambique—genotypes II, V, and VI (*12*).

The genotype II Madagascar isolate, MAD 1/98, was obtained from a domestic pig in 1998 during the first outbreak of ASF that affected the island country. The more recent Madagascar pig isolates obtained in 1999–2003 are presumed to have derived from this first introduction because they belong to the same genotype. Mozambique has been speculated to be the most likely source of infection for the 1998 ASFV outbreaks occurring in Madagascar because the isolates from Mozambique were genotype II and identical across the *B602L* region (*22*). Before 1998, the island of Madagascar was free of the disease (*21*,*22*). The genotype II isolate from Zambia (Lus 1/93) was isolated from an infected domestic pig in 1991, whereas the viruses from Mozambique were isolated from domestic pigs during outbreaks of the disease along the eastern coast of the country in 2002–2005.

Further sequence analysis was performed on 2 other conserved regions of the ASFV genome; the ORFs *E183L* and *CP204L*, which encode the structural proteins p54 and p30, respectively. Analysis of the *E183L* gene showed that the Georgia 2007 isolates were most closely related to 4 isolates from Madagascar, which were in circulation in 1999–2003, and 2 isolates from Mozambique but distinguishable from the group II isolate Lus 1/93 and the Mozambique isolates Moz 1/03 and Moz 1/05. Similarly, analysis of the *CP204L* gene encoding p30 showed the Georgia 2007 isolates were distinguishable from all other isolates, although they were most closely related to the 4 isolates from Madagascar in circulation in 1999–2003, the Zambian Lus 1/93 isolate, and one of the Mozambique isolates (2/02).

Fragment size analysis has identified *B602L* as the most variable genome region (*15*). The variable region of *B602L* contains amino acid tetramers that vary in number and type. Sequence analysis of the *B602L* gene from the Georgian isolates identified 5 different amino acid tetramer sequences encoded in this genome region. One of these tetramer sequences was CTST, which is one of the less common tetramer sequences (*15*). The sequence of the *B602L* variable region from the Georgian isolate grouped it with isolates in circulation in Madagascar (1999–2003) as well as isolates from Mozambique from outbreaks in 1960, 1961, 1963, 1970, and 1998.

The first case of ASF in Georgia was observed in the Samegrelo region on the west coast, which suggests a possible connection to the port of Poti on the Black Sea. One possibility is that that the virus entered Georgia through meat products since ASFV may remain viable for long periods in infected pig tissues, meat, and processed pig products. Most pigs in Georgia are kept on a free-ranging, scavenging system, and so access to or swill feeding of dumped port waste is possible. However, several events would be required to cause an outbreak, making this a relatively rare event and providing an explanation for the relatively few incidents of transcontinental spread of ASFV. Our analysis showed that the Georgia strain is most similar to isolates from Madagascar. However, since few ASFV samples are submitted for genotyping, it is possible that viruses belonging to genotype II may be more widespread. However, it seems likely that the source of infection of the Georgia 2007 outbreak is from the eastern side of southern Africa or Madagascar rather than west or central Africa or Sardinia.

## Supplementary Material

Appendix Table Summary of African swine fever isolates obtained from 1960 through 2007 and compared in this study

Appendix Figure 1 Amino acid sequence alignment of the central variable region of B602L from different African swine fever virus isolates previously identified in genotypes II, V, VI, VIII, XX, XIX, and XXI. All the sequences of the Georgian isolates were identical; only one is shown. Amino acids are arranged as tetrameric repeats. Indels are indicated by dashes.

Appendix Figure 2 Sequence comparison of E183L sequences of African swine fever virus isolates from genotype II. A DNA sequence alignment of the E183L open reading frame from Genotype II isolates is shown. Deduced amino acid translations are shown above the nucleotide sequences. Dots indicate nucleotides identical to that of the sequence Georgia 2007. Nucleotides that differ from this sequence are in boldface. Indels are indicated by dashes. Asterisks indicate regions of sequence identical across all isolates shown.

Appendix Figure 3 Sequence comparison of the CP204L gene encoding protein p30 from genotype II of African swine fever virus. A DNA sequence alignment of the CP204L ORF from genotype II isolates is represented, with the corresponding amino acid translation shown above. Dots indicate nucleotides identical to that of the master sequence Georgia 2007. Nucleotides that differ from the master sequence are in boldface; changes to the amino acid sequence are illustrated above the site of change. Asterisks indicate identical regions of sequence across all isolates shown. -, a gap inserted for alignment purposes. Alignments were performed by using ClustalW (1.83) software (www.clustal.org).
