# Supplementary material for: African Swine Fever Virus Isolate, Georgia, 2007
Source: Emerg Infect Dis. 2008 Dec;14(12):1870–4. doi: 10.3201/eid1412.080591 (PMC2634662; doi:10.3201/eid1412.080591)
Supplement: Appendix Table — Summary of African swine fever isolates obtained from 1960 through 2007 and compared in this study [file 08-0591_appT-s1.pdf]

Publisher: CDC; Journal: Emerging Infectious Diseases
Article Type: Research; Volume: 14; Issue: 12; Year: 2008; Article ID: 08-0591
DOI: 10.3201/eid1412.080591; TOC Head: Research

Appendix Table. Summary of African swine fever isolates obtained from 1960 through 2007 and compared in this study

Isolate	Geographic origin	Year	Species	*B646L* GenBank accession no.	Reference	*B646L* genotype	*E183L* GenBank accession no.	Reference	*CP204L* GenBank accession no.	Reference	*B602L* GenBank accession no.	Reference
Georgia/2007	Georgia	2007	Domestic pig	AM999764	This study	II	AM999765	This study	AM999766	This study	AM999767	This study
Bots 1/99	Botswana	1999	Domestic pig	AF504886	(*3*)	III	–	–	–	–	–	-
Mad 1/98	Madagascar	1998	Domestic pig	AF270706	(*3*)	II	–	–	–	–	AY274471	(*3*)
Ampani/99	Madagascar	1999	Domestic pig	–	–	II	EU62068	This study	EU620686	This study	EU649698	This study
Tolagna/99	Madagascar	1999	Domestic pig	–	–	II	EU620683	This study	EU620689	This study	-	-
Chrome/01	Madagascar	2001	Domestic pig	–	–	II	EU620682	This study	EU620688	This study	EU649697	This study
Antani/03	Madagascar	2003	Domestic pig	–	–	II	EU620681	This study	EU620687	This study	EU649696	This study
Ten/60	Malawi	1960	Domestic pig	AF301541	(*3*)	V	–	–	–	–	AM259417	(*15*)
Moz 1/94	Mozambique	1994	Domestic pig	AF270711	(*3*)	VI	–	–	–	–	AY274468	(*11*)
Moz 1/01	Mozambique	2001	Domestic pig	AY351516	(*5*)	VIII	–	–	–	–	DQ874377	(*5*)
Moz 1/02	Mozambique	2002	Domestic pig	AY351517	(*5*)	II	EU874380	This study	EU874315	This study	AY351517	This study
Moz 2/02	Mozambique	2002	Domestic pig	AY351518	(*5*)	II	EU874376	This study	EU874274	This study	AY351518	This study
Moz 1/03	Mozambique	2003	Domestic pig		This study	II	EU874379	This study	EU874314	This study	Submitted	This study
Moz 1/05	Mozambique	2005	Domestic pig		This study	II	EU874378	This study	EU874313	This study	Submitted	This study
Nam 1/95	Namibia	1995	Domestic pig	DQ250122	(*4*)	XVIII	–	–	–	–	–	-
Lis/57	Portugal	1957	Domestic pig	AF301537	(*3*)	I	–	–	–	–	–	-
Spec /245	South Africa	1992	Domestic pig	DQ250117	(*4*)	XXII	–	–	–	–	–	-
RSA 1/95	South Africa	1995	Domestic pig	DQ250123	(*4*)	XX	–	–	–	–	DQ250101	(*4*)
RSA 1/96	South Africa	1996	Domestic pig	DQ250125	(*4*)	XXI	–	–	–	–	DQ250103	(*4*)
RSA 2/96	South Africa	1996	Domestic pig	DQ250126	(*4*)	XIX	–	–	–	–	DQ250104	(*4*)
RSA 1/98	South Africa	1998	Domestic pig	AF302818	(*3*)	VII	–	–	–	–	DQ250106	(*4*)
RSA 1/99	South Africa	1999	Warthog	AF449477	(*3*)	IV	–	–	–	–	–	-
Tan 1/01	Tanzania	2001	Domestic pig	AY494552	(*5*)	XV	–	–	–	–	–	-
Tan 1/03	Tanzania	2003	Domestic pig	AY494550	(*5*)	XVI	–	–	–	–	–	-
Uga 1/95	Uganda	1995	Domestic pig	AF449475	(*3*)	IX	–	–	–	–	–	-
Uga 1/93	Uganda	1995	Domestic pig	AF449476	(*3*)	X	–	–	–	–	–	-
MFUE 6/1	Zambia	1982	Tick	AY351561	(*5*)	XII	–	–	–	–	–	-
Kab/62	Zambia	1983	Tick	AY351522	(*5*)	XI	–	–	–	–	–	-
Sum 14/11	Zambia	1983	Tick	AY351542	(*5*)	XIII	–	–	–	–	–	-
NYA 1/2	Zambia	1986	Tick	AY351555	(*5*)	XIV	–	–	–	–	–	-
Lus 1/93	Zambia	1991	Domestic pig	AY351563	(*5*)	II	–	–	–	–	–	-
Zim 1/92	Zimbabwe	1992	Domestic pig	DQ250119	(*4*)	XVII	–	–	–	–	–	-
