# Supplementary material for: African Swine Fever Virus Isolate, Georgia, 2007
Source: Emerg Infect Dis. 2008 Dec;14(12):1870–4. doi: 10.3201/eid1412.080591 (PMC2634662; doi:10.3201/eid1412.080591)
Supplement: Appendix Figure 1 — Amino acid sequence alignment of the central variable region of B602L from different African swine fever virus isolates previously identified in genotypes II, V, VI, VIII, XX, XIX, and XXI. All the sequences of the Georgian isolates were identical; only one is shown. Amino acids are arranged as tetrameric repeats. Indels are indicated by dashes. [file 08-0591_appF1-s2.pdf]

Georgia/2007	CADT	----	----	----	----	----	----	----	----	----	----	----	----	NVDT	CASM	CADT	----	NVDT	CASM	----	CADT	NVDT	CAST	CTST	II
Mad 1/98[2]	CADT	----	----	----	----	----	----	----	----	----	----	----	----	NVDT	CASM	CADT	----	NVDT	CASM	----	CADT	NVDT	CAST	CTST	II
Ampani /99[1]	CADT	----	----	----	----	----	----	----	----	----	----	----	----	NVDT	CASM	CADT	----	NVDT	CASM	----	CADT	NVDT	CAST	CTST	II
Chrome /01[1]	CADT	----	----	----	----	----	----	----	----	----	----	----	----	NVDT	CASM	CADT	----	NVDT	CASM	----	CADT	NVDT	CAST	CTST	II
Antani /03[1]	CADT	----	----	----	----	----	----	----	----	----	----	----	----	NVDT	CASM	CADT	----	NVDT	CASM	----	CADT	NVDT	CAST	CTST	II
MOZ 1/02	CADT	----	----	----	----	----	----	----	----	----	----	----	----	NVDT	CASM	CADT	----	NVDT	CASM	----	CADT	NVDT	CAST	CTST	II
MOZ 2/02	CADT	----	----	----	----	----	----	----	----	----	----	----	----	NVDT	CASM	CADT	----	NVDT	CASM	----	CADT	NVDT	CAST	CTST	II
MOZ 1/03	CADT	----	----	----	----	----	----	----	----	----	----	----	----	NVDT	CASM	CADT	----	NVDT	CASM	----	CADT	NVDT	CAST	CTST	II
MOZ 1/05	CADT	----	----	----	----	----	----	----	----	----	----	----	----	NVDT	CASM	CADT	----	NVDT	CASM	----	CADT	NVDT	CAST	CTST	II
Ten/60[2]	CAST	CADT	NVDT	CAST	CAST	CAST	CAST	CTST	----	CADT	NVDT	CADT	----	NVDT	CADT	CAST	CADT	NVDT	CADT	CAST	CADT	NEDT	CAST	CTST	V
MOZ 1/94[2]	CAST	CAST	CADT	CAST	----	----	----	----	----	CADT	NVDT	CAST	----	----	CADT	CAST	CADT	NVDT	CADT	CAST	CADT	NEDT	CAST	----	VI
RSA 1/98	CADT		NAST	SADT	NAST	NADT	----	----	----	----	NVGT	----	----	NVGT	CAST	CAST	CAST	----	CAST	CAST	----	----	CAST	CANT	VII
MOZ 1/01	CAST	----	NADT	SAST	NADT	SAST	----	----	NASI	NADT	NVDT	CAST	NAST	NVDT	NASI	NAST	NAST	NVDT	NAST	NADI	NANT	NADI	NANI	NANT	VIII
RSA 1/95[4]	CADT	----	NAST	SADT	NAST	SADT	----	----	NAST	NADT	NVGT	CAST	----	----	----	----	----	----	----	CAST	CADT	----	CAST	CTNT	XX
RSA 2/96[4]	CADT	----	----	----	NAST	SADT	NAST	SADT	----	----	----	----	----	----	----	NAST	NADT	NVGT	CAST	CAST	CADT	----	CAST	CTNT	XIX
RSA 1/96[4]	CAST	----	----	----	----	----	NAST	SADT	----	----	----	----	----	NVGT	----	CAST	----	----	----	CAST	NADT	NVNT	CAST	CTST	XXI
