# Supplementary material for: African Swine Fever Virus Isolate, Georgia, 2007
Source: Emerg Infect Dis. 2008 Dec;14(12):1870–4. doi: 10.3201/eid1412.080591 (PMC2634662; doi:10.3201/eid1412.080591)
Supplement: Appendix Figure 2 — Sequence comparison of E183L sequences of African swine fever virus isolates from genotype II. A DNA sequence alignment of the E183L open reading frame from Genotype II isolates is shown. Deduced amino acid translations are shown above the nucleotide sequences. Dots indicate nucleotides identical to that of the sequence Georgia 2007. Nucleotides that differ from this sequence are in boldface. Indels are indicated by dashes. Asterisks indicate regions of sequence identical across all isolates shown. [file 08-0591_appF2-s3.pdf]

```
                 M  D  S  E  F  F  Q  P  V  Y  P  R  H  Y  G  E  C  L  S  P
Georgia/07       ATGGATTCTGAATTTTTTCAACCGGTTTATCCGCGGCATTATGGTGAGTGTTTGTCACCA 60
Tolagna/99       ............................................................ 60
Lus 1/93         ............................................................ 60
Ampani/99        ............................................................ 60
Chrome/01        ............................................................ 60
Antani/03        ............................................................ 60
Moz 1/02         ............................................................ 60
Moz 1/03         ............................................................ 60
Moz 1/05         ............................................................ 60
Moz 2/02         ............................................................ 60


                 V  T  T  P  S  F  F  S  T  H  M  Y  T  I  L  I  A  I  V  V
Georgia/07       GTCACTACACCAAGCTTCTTCTCCACACATATGTATACTATTCTCATTGCTATCGTGGTC 120
Tolagna/99       ............................................................ 120
Lus 1/93         ............................................................ 120
Ampani/99        ............................................................ 120
Chrome/01        ............................................................ 120
Antani/03        ............................................................ 120
Moz 1/02         ............................................................ 120
                    S
Moz 1/03         ...T........................................................ 120
                    S
Moz 1/05         ...T........................................................ 120
Moz 2/02         ............................................................ 120
```

```
                 L  V  I  I  I  I  V  L  I  Y  L  F  S  S  R  K  K  K  A  A
Georgia/07       TTAGTCATCATTATCATCGTTCTAATCTATCTATTCTCTTCAAGAAAGAAAAAAGCTGCT 180
Tolagna/99       ............................................................ 180
Lus 1/93         ............................................................ 180
Ampani/99        ............................................................ 180
Chrome/01        ............................................................ 180
Antani/03        ............................................................ 180
Moz 1/02         ............................................................ 180
Moz 1/03         ........T.................T........T........................ 180
Moz 1/05         ........T.................T........T........................ 180
Moz 2/02         ............................................................ 180


                       A  I  E  E  E  D  I  Q  F  I  N  P  Y  Q  D  Q  Q  W
Georgia/07       ------GCTATTGAGGAGGAAGATATACAGTTTATAAATCCTTATCAAGATCAGCAGTGG 234
Tolagna/99       ------...................................................... 234
Lus 1/93         ------...................................................... 234
Ampani/99        ------...................................................... 234
Chrome/01        ------...................................................... 234
Antani/03        ------...................................................... 234
Moz 1/02         ------...................................................... 234
                 A
Moz 1/03         G---CC........A............................................. 237
                 A
Moz 1/05         G---CC........A............................................. 237
Moz 2/02         ------...................................................... 234
```

```
                V  E  V  T  P  Q  P  G  T  S  K  P  A  G  A  T  T  A  S  V
Georgia/07      GTAGAAGTCACTCCACAACCAGGTACCTCTAAACCAGCTGGAGCGACTACAGCAAGTGTA 294
Tolagna/99      ............................................................ 294
Lus 1/93        ............................................................ 294
Ampani /99      ............................................................ 294
Chrome /01      ............................................................ 294
Antani /03      ............................................................ 294
Moz 1/02        ............................................................ 294
                                                          V
Moz 1/03        ...................................G.......T................ 297
                                                          V
Moz 1/05        ...................................G.......T................ 297
Moz 2/02        ............................................................ 294


                G  K  P  V  T  G  R  P  A  T  N  A  P  A  T  N  K  P  V  T
Georgia/07      GGCAAGCCAGTCACGGGCAGACCGGCAACAAACAGACCAGCAACAAACAAACCAGTTACG 354
Tolagna/99      ............................................................ 354
Lus 1/93        ..............................................-------------- 340
Ampani/99       ............................................................ 354
Chrome/01       ............................................................ 354
Antani/03       ............................................................ 354
Moz 1/02        ............................................................ 354
                                                       I  T  D  R  L  V  M
Moz 1/03        ....................G...................TT..GG...GG.T...C.T. 357
                                                       I  T  D  R  L  V  M
Moz 1/05        ....................G...................TT..GG...GG.T..TC.T. 357
Moz 2/02        ............................................................ 354
```

```
                D  N  P  V  T  D  R  L   V  M  A  T  G  G   P
Georgia/07      GACAACCCAGTTACGGACAGAC-TAGTCATGGCAACTGGCG-GGCCG-------------- 399
Tolanga/99      ......................-..................-.....-------------- 399
Lus 1/93        -.....................-..................-.....-------------- 385
Ampani/99       ......................-..................-.....-------------- 399
Chrome/01       ......................-..................-.....-------------- 399
Antani/03       ......................-..................-.....-------------- 399
Moz 1/02        ......................-..................-.....-------------- 399
                A   T  G  G  P  A  A  A  S  A  A  A  S  A  A  T  N  T  A  A
Moz 1/03        .-....TGGCGGG.CAG.G.C.GC.AGTGC...CG.AA.T.C....ACGAATACAGCTGCG 417
                A   T  G  G  P  A  A  A  S  A  A  A  S  A  A  T  N  T  A  A
Moz 1/05        .-....TGGCGGG.CAG.G.C.GC.AGTGC...CG.AA.T.C....ACGAATACAGCTGCG 417
Moz 2/02        .....................-...................-.....-------------- 399
                  ****       *   * * *  *     ***  *  * * ****


                   A  A  A  P  A  A  A  S  A  P  A  H  P  A  E  P  Y  T  T
Georgia/07      ---GCGGCCGCACCTGCGGCCGCGAGTGCTCCTGCTCATCCGGCTGAGCCTTACACGACA 455
Tolagna/99      ---......................................................... 455
Lus 1/93        ---......................................................... 440
Ampani/99       ---......................................................... 455
Chrome/01       ---......................................................... 455
Antani/03       ---......................................................... 455
Moz 1/02        ---......................................................... 455
                S           S
Moz 1/03        AGT..A.....GAG...A...................................T..... 476
                S           S
Moz 1/05        AGT..A.....GAG...A...................................T..... 476
Moz 2/02        ---........................................................ 455
```

```
                V  T  T  Q  N  T  A  S  Q  T  M  S  A  I  E  N  L  R  Q  R
Georgia/07      GTCACTACTCAGAACACTGCTTCACAAACAATGTCGGCTATTGAAAATTTACGACAAAG 515
Tolagna/99      ........................................................... 515
Lus 1/93        ........................................................... 500
Ampani/99       ........................................................... 515
Chrome/01       ........................................................... 515
Antani/03       ........................................................... 515
Moz 1/02        ........................................................... 515
Moz 1/03        .......................C........................C....G..... 536
Moz 1/05        .......................C........................C....G..... 536
Moz 2/02        ........................................................... 515


                 N  T  Y  T  H  K  D  L  E  N  S  L  *
Georgia/07      AAACACCTATACGCATAAAGACCTAGAAAACTCCTTGTAA 555
Tolagna/99      ........................................ 555
Lus 1/93        ........................................ 540
Ampani/99       ........................................ 555
Chrome/01       ........................................ 555
Antani/03       ........................................ 555
Moz 1/02        ........................................ 555
Moz 1/03        ..G..................................... 576
Moz 1/05        ..G..................................... 576
Moz 2/02        ........................................ 555
```
