# Supplementary material for: African Swine Fever Virus Isolate, Georgia, 2007
Source: Emerg Infect Dis. 2008 Dec;14(12):1870–4. doi: 10.3201/eid1412.080591 (PMC2634662; doi:10.3201/eid1412.080591)
Supplement: Appendix Figure 3 — Sequence comparison of the CP204L gene encoding protein p30 from genotype II of African swine fever virus. A DNA sequence alignment of the CP204L ORF from genotype II isolates is represented, with the corresponding amino acid translation shown above. Dots indicate nucleotides identical to that of the master sequence Georgia 2007. Nucleotides that differ from the master sequence are in boldface; changes to the amino acid sequence are illustrated above the site of change. Asterisks indicate identical regions of sequence across all isolates shown. -, a gap inserted for alignment purposes. Alignments were performed by using ClustalW (1.83) software (www.clustal.org). [file 08-0591_appF3-s4.pdf]

```
                   M  K  M  E  V  I  F  K  T  D  L  R  S  S  S  Q  V  V  F  H
Georgia/07         ATGAAAATGGAGGTCATCTTCAAAACGGATTTAAGATCATCTTCACAAGTTGTGTTTCAT 60
Ampani/99          ............................................................
Antani/03          ............................................................
Chrome/01          ............................................................
Tolagna/99         ............................................................
Lus 1/93           ............................................................
Moz 1/03           ............................................................
Moz 1/05           ............................................................
Moz 1/02           ............................................................
Moz 2/02           ............................................................

                   A  G  S  L  Y  N  W  F  S  V  E  I  I  N  S  G  R  I  V  T
Georgia/07         GCGGGTAGCCTGTATAATTGGTTTTCTGTTGAGATTATCAATAGCGGTAGAATTGTTACG 120
Ampani/99          ............................................................
Antani/03          ............................................................
Chrome/01          ............................................................
Tolagna/99         ............................................................
Lus 1/93           ............................................................
Moz 1/03           ..A.........................................................
Moz 1/05           ..A.........................................................
Moz 1/02           ............................................................
Moz 2/02           ............................................................
```

```
                    T  A  I  K  T  L  L  S  T  V  K  Y  D  I  V  K  S  A  R  I
Georgia/07          ACCGCTATAAAAACATTGCTTAGTACTGTTAAGTATGATATTGTGAAATCTGCTCGTATA 180
Ampani/99           ............................................................
Antani/03           ............................................................
Chrome/01           ............................................................
Tolagna/99          ............................................................
Lus 1/93            .....................C......................................
Moz 1/03            .....................C......................................
Moz 1/05            .....................C......................................
Moz 1/02            ............................................................
Moz 2/02            ............................................................
```

```
                    Y  A  G  Q  G  Y  T  E  H  Q  A  Q  E  E  W  N  M  I  L  H
Georgia/07          TATGCAGGGCAAGGGTATACTGAACATCAGGCTCAAGAAGAATGGAATATGATTCTGCAT 240
Ampani/99           ............................................................
Antani/03           ............................................................
Chrome/01           ............................................................
Tolagna/99          ............................................................
Lus 1/93            ............................................................
Moz 1/03            ........A...................................................
Moz 1/05            ............................................................
Moz 1/02            ............................................................
Moz 2/02            ............................................................
```

```
                   V  L  F  E  E  E  T  E  S  S  A  S  S  E  N  I  H  E  K  N
Georgia/07         GTGCTGTTTGAAGAGGAGACGGAATCCTCAGCATCTTCGGAGAACATTCATGAAAAAAAT 300
Ampani/99          ............................................................
Antani/03          ............................................................
Chrome/01          ............................................................
Tolagna/99         ...T........................................................
                                                              S
Lus 1/93           ...................................A.......G................
Moz 1/03           ..................A................A.....A..................
Moz 1/05           ..................A................A.....A..................
Moz 1/02           ............................................................
                                                              S
Moz 2/02           ...................................A.....A.G................


                   D  N  E  T  N  E  C  T  S  S  F  E  T  L  F  E  Q  E  P  S
Georgia/07         GATAATGAAACCAATGAATGCACATCCTCCTTTGAAACGTTGTTTGAGCAAGAGCCCTCA 360
Ampani/99          ............................................................
Antani/03          ............................................................
Chrome/01          ............................................................
Tolagna/99         ............................................................
Lus 1/93           ............................................................
                                                                          P
Moz 1/03           ......................................A..................C..
Moz 1/05           ......................................A..................C..
Moz 1/02           ......................................A..................C..
Moz 2/02           ............................................................
```

```
                    S  E  V  P  K  D  S  K  L  Y  M  L  A  Q  K  T  V  Q  H  I
Georgia/07          TCGGAGGTACCTAAAGACTCCAAGCTGTATATGCTTGCACAAAAGACTGTGCAACATATT 420
                                                        V
Ampani/99           .....................................T......................
Antani/03           ............................................................
Chrome/01           ............................................................
Tolagna/99          ............................................................
Lus 1/93            ............................................................
                       D  A              T
Moz 1/03            ..A..T.C..............C.................................C...
                       D  A              T
Moz 1/05            ..A..T.C..............C.................................C...
                       D  A              T
Moz 1/02            ..A..T.C..............C.................................C...
Moz 2/02            ............................................................


                    E  Q  Y  G  K  A  P  D  F  N  K  V  I  R  A  H  N  F  I  Q
Georgia/07          GAACAATATGGAAAGGCACCTGATTTTAACAAGGTTATTAGAGCACATAATTTTATTCAA 480
Ampani/99           ............................................................
Antani/03           ............................................................
Chrome/01           ............................................................
Tolagna/99          ............................................................
                                                                       L
Lus 1/93            ...................................................C........
Moz 1/03            ...................................................C........
Moz 1/05            ...................................................C........
Moz 1/02            ...................................................C........
Moz 2/02            ...................................................C........
```

T I Y G T P L K E E E K E V V R L M V I

Georgia/07 ACCATTTATGGAACCCCTCTAAAGGAAGAAGAAAAAGAGGTGGTAAGACTCATGGTCATT 540

Ampani/99 ....**T**...

Antani/03 ....**T**...

Chrome/01 ....**T**...

Tolagna/99 ....**T**...

Lus 1/93 ....**T**...

S

Moz 1/03 ....**C**..

S

Moz 1/05 ....**C**..

S

Moz 1/02 ....**C**..

Moz 2/02 ....**T**...

K L

Georgia/07 AAACTT 545

Ampani/99 ......

Antani/03 ......

Chrome/01 ......

Tolagna/99 ......

Moronda02 ......

Lus 1/93 ......

Moz 1/03 ......

Moz 1/05 ......

Moz 1/02 ......

Moz 2/02 ......
